# Editorial: The mechanistic investigation and emerging therapies for Friedreich’s ataxia

**DOI:** 10.3389/fphar.2025.1560808

**Published:** 2025-02-10

**Authors:** Yina Dong, Vijayendran Chandran, Elisabetta Soragni, David R. Lynch

**Affiliations:** ^1^ Department of Pediatrics and Neurology, The Children’s Hospital of Philadelphia, Philadelphia, PA, United States; ^2^ Department of Neurology, Perelman School of Medicine, University of Pennsylvania, Philadelphia, PA, United States; ^3^ Department of Pediatrics, University of Florida, Gainesville, FL, United States; ^4^ Friedreich’s Ataxia Research Alliance, Downingtown, PA, United States

**Keywords:** gene - editing, frataxin regulation, antioxidant therapy, mitochondrial dysfunction, oxidative stress, Friedreich’s Ataxia

Friedreich’s ataxia (FRDA) is a multisystem disorder caused by a GAA repeat expansion in the first intron of the *FXN* gene. This genetic mutation reduces the transcription of *FXN* mRNA, leading to a deficiency in frataxin protein, a key regulator of mitochondrial function. Frataxin loss disrupts mitochondrial homeostasis, resulting in cellular energy deficits and oxidative stress, which manifest in progressive pathology across the cerebellum, skeletal muscle, and heart. Clinically, FRDA presents with gait and limb ataxia, dysarthria, scoliosis, and cardiomyopathy—the latter being the leading cause of death. Disease progression is relentless, often leading to wheelchair dependence within 10–15 years of onset. Despite the severity of FRDA, there is currently no cure, and Omaveloxolone remains the only FDA- and EMA-approved treatment, specifically for patients aged 16 years and older. However, given the multisystemic nature of the disease, effective long-term treatment will likely require a combinatorial approach targeting multiple tissues and symptoms. This underscores the importance of continued efforts to elucidate frataxin regulation, identify strategies to restore frataxin levels, and develop sensitive biomarkers and clinical endpoints for trials. This Research Topic brings together six studies that advance the understanding of FRDA pathophysiology and introduce promising therapeutic strategies and outcome measures.

Several lines of evidence from both animal models and patients suggest that skeletal muscle plays a significant role in FRDA pathology. Muscle dysfunction can contribute to fatigue and reduced exercise tolerance, both of which are commonly experienced by FRDA patients. Beyond its involvement in disease symptoms, skeletal muscle is an accessible tissue, making it a promising candidate for both therapeutic targeting and the identification of surrogate biomarkers for clinical trials. In this context, Indelicato et al. employed a skeletal muscle-specific proteomic analysis, identifying 228 differentially expressed mitochondrial proteins in FRDA patients. Their findings confirmed the well-established disruption of oxidative phosphorylation—a hallmark of FRDA-but also revealed previously unrecognized mitochondrial architectural abnormalities and disturbances in maintenance pathways, such as defective mitochondrial fission/fusion dynamics and a metabolic shift toward glycolytic fibers. These insights not only deepen our understanding of mitochondrial dysfunction in FRDA but also underscore the relevance of skeletal muscle as a potential readout for disease state and response to therapy.

Upregulating frataxin expression remains a critical therapeutic strategy for FRDA, and gene editing technologies offer a direct approach to correct the underlying genetic defect. Mishra et al. explored the potential of CRISPR/Cas9 gene editing to address the GAA repeat expansion in *FXN*, which directly drives frataxin deficiency. Using FRDA patient-derived induced pluripotent stem cell (iPSC) neurons, they demonstrated that gene correction successfully restored frataxin levels and improved mitochondrial health, as evidenced by reduced apoptosis, oxidative stress, and mitochondrial damage. Additionally, the study revealed novel insights into FRDA pathophysiology, identifying abnormalities in endoplasmic reticulum (ER) morphology and compromised ER-mitochondrial contacts—both of which were restored following gene editing. These findings not only validate gene editing as a potential therapeutic approach but also uncover previously unexplored cellular mechanisms contributing to FRDA pathology, emphasizing the importance of targeting both frataxin restoration and cellular homeostasis.

In a complementary approach, Dong et al. investigated a small peptide-based strategy for frataxin upregulation by targeting a protein interaction regulator. They identified a six-amino acid peptide, TID1S448-453, derived from the mitochondrial J-protein cochaperone TID1S. This peptide was designed based on the discovery that TID1S physically interacts with and negatively regulates frataxin levels. By disrupting the frataxin-TID1S interaction, the peptide successfully increased frataxin levels and improved mitochondrial function in FRDA patient fibroblasts. The low molecular weight and modifiability of the TID1S448-453 peptide make it an attractive candidate for therapeutic development, particularly as an alternative approach for restoring frataxin levels. These findings open a promising new avenue for therapy design, emphasizing the potential of targeting protein interactions to correct mitochondrial dysfunction in FRDA.

Accurate measurement of disease progression and functional decline in FRDA is crucial for both clinical care and the development of effective therapies. Fichera et al. addressed this need by exploring the use of wearable sensor technology to monitor physical activity in FRDA patients over a 1-year period. Using accelerometers to capture real-life activity, the study provided an objective and sensitive assessment of physical functioning beyond standard clinical scales. Their data revealed a significant decline in physical activity over the study period, correlating closely with established clinical measures such as the SARA gait sub-score and disease duration. Notably, the technology allowed for continuous monitoring without limiting patients to specific tasks or clinical settings, capturing broader aspects of daily life. These findings support the use of wearable technologies as reliable tools for assessing functional decline, providing critical endpoints for future FRDA trials aimed at measuring treatment effects and quality of life.

Oxidative stress plays a central role in FRDA pathology and has been a key focus for therapeutic development. Edzeamey et al. reviewed the landscape of antioxidant therapies aimed at counteracting the oxidative damage associated with frataxin deficiency. Their comprehensive analysis explored both direct reactive oxygen species (ROS) scavengers and agents targeting mitochondrial pathways, such as Nrf2 activators dimethyl fumarate (DMF) and omaveloxolone. While omaveloxolone has emerged as the first FDA- and EMA-approved treatment for FRDA, the authors emphasized the limitations of single-agent therapies due to the multifactorial nature of oxidative stress in FRDA. They proposed that combination therapies targeting multiple pathways may offer greater efficacy by producing synergistic effects. The positive clinical trial results with omaveloxolone thus far reinforce the significance of oxidative stress in FRDA and suggest a promising avenue for further therapeutic exploration using antioxidant strategies.

Drug repurposing has also emerged as a promising strategy for FRDA treatment, leveraging existing compounds to accelerate clinical translation. Pane et al. explored the potential of dimethyl fumarate (DMF), an FDA-approved treatment for multiple sclerosis, based on its ability to activate the Nrf2 pathway and increase frataxin levels in FRDA models. In an exploratory study, DMF treatment in multiple sclerosis patients led to a significant elevation in *FXN* mRNA levels compared to controls. Building on this evidence, the authors designed a double-blind, placebo-controlled trial to assess the safety, efficacy, and tolerability of DMF in FRDA patients. The primary endpoint of this trial is a change in *FXN* gene expression, with secondary endpoints including frataxin protein levels, cardiopulmonary exercise test results, echocardiographic measures, and clinical scales. This study will not only test DMF as a potential treatment but also provide insights into the utility of novel biomarkers and functional outcomes for future FRDA trials.

Collectively, the six studies presented in this Research Topic significantly advance our understanding of FRDA pathophysiology, therapeutic targets, and outcome measures. They underscore the importance of mitochondrial dysfunction, oxidative stress, and frataxin regulation in disease progression, while also introducing innovative therapeutic strategies, such as gene editing, small peptide modulators, and antioxidant approaches. Furthermore, the use of wearable technologies and accessible tissues like skeletal muscle highlights new avenues for biomarker discovery and improved clinical trial endpoints. As the field moves forward, these findings emphasize the need for multifaceted treatment strategies targeting multiple pathways and tissues to effectively manage FRDA. By integrating mechanistic insights with novel therapeutic approaches and outcome measures, this collection paves the way for more comprehensive and effective interventions for FRDA patients.

